# An Interdisciplinary Approach for Compulsive Behavior in Dogs: A Case Report

**DOI:** 10.3389/fvets.2022.801636

**Published:** 2022-03-24

**Authors:** Danila d'Angelo, Luigi Sacchettino, Rosanna Carpentieri, Luigi Avallone, Claudia Gatta, Francesco Napolitano

**Affiliations:** ^1^Department of Veterinary Medicine and Animal Production, University of Naples Federico II, Naples, Italy; ^2^Veterinary Practitioner, Salerno, Italy; ^3^CEINGE-Biotecnologie Avanzate, Naples, Italy

**Keywords:** dog behavior, obsessive-compulsive disorder (OCD), tail chasing, human-dog relationship, fluoxetine

## Abstract

Compulsive disorder is a debilitating condition affecting both humans and animals, characterized by intrusive thoughts and recurring out-of-place behaviors. Among them, tail chasing might represent one of the most common traits in compulsive dogs. Herein, we reported the case of a 7-year-old intact male German Shepherd mixed-breed dog, presenting with tail chasing behavior. He underwent a first behavioral evaluation 1 year before (at the age of 6), when he injured himself with severe wounds at level of the tail and left thigh. To avoid any specific suffering and increase his physical health, of course, the study was carried out through an interdisciplinary approach, employing a veterinary behaviorist and a rehabilitating dog instructor. Three months after pharmacological treatment with fluoxetine and α-s1 casozepine, associated with a behavioral recovery program, the owner reported an improvement of compulsive events in his dog, in terms of intensity and frequency. Interestingly, over the following 3 months, the dog did not experience any new tail chasing episodes.

## Introduction

Like in humans, the obsessive compulsive disorder (OCD) in pet dogs and cats (hereafter CD) is characterized by a constant and time-consuming repetition of behaviors, emancipated from the environment, that appear to serve no obvious purposes, for which they are generally referred to as stereotypies (1). In dogs, CD include circling, barking, fly biting, chewing on toys, self-mutilation, and acral lick dermatitis, which definitely affect their everyday life. These out-of-context activities are generally due to conflict or frustration, increasing anxiety state, that normally appear when animals cannot cope with stressful situations, thus eventually overcoming the “critical” behavioral threshold. Notably, previous findings documented that about 50% of CD dogs respond to the antidepressant clomipramine, found to improve the “obsessive” symptom in human patients (2, 3). Among genetically isolated dog populations, Bull Terriers, Doberman Pinschers, and German Shepherds were shown to have a high rate to develop CD pathology (1, 4, 5). The age of occurrence for such a psychiatric disorder in dogs may mirror that experienced in human patients, showing up at the peri-pubertal phase (6–12 months of age), thus suggesting a potential genetic liability of such a disorder (5, 6). Tail chasing (TC) is a classic compulsive behavior experienced by dogs, often occurring in phasic fashion, characterized by episodes where the dog stares at its tail quietly for a while, before resuming chasing. Maternal deprivation was shown to even contribute to the development of similar stereotypic behaviors in both humans and dogs. Accordingly, Grisham and colleagues substantiated that childhood trauma and stressful events are associated with human OCD (6). Similarly, a recent study performed on a pet population of 368 dogs from four dog breeds displayed that TC animals, separated earlier from their mothers, experienced a lower-quality care from their mothers, associated with anxiety-related behaviors (7).

## Case Description

We reported the case of a 7-year-old intact male, mixed-breed German Shepherd, 40 kg in weight, who underwent behavioral examination at the age of 6, because of lacerated lesions both in the tail and in the region of the left thigh, caused by the TC symptom. The patient was referred to a veterinary behaviorist and a dog instructor, both of them working in the clinic where he was assisted in ECC (emergency critical care). The following day, they took charge in managing him for the behavioral-related evaluation. As also reported in the behavioral timeline (Table 1), he was found by a volunteer on the street, together with five other 20-day-old puppies, who kept him in a stall, without any kind of contact with dogs, people, and the environment, until he was adopted at 4 months of age. He would live in a family consisting of an elderly mother, who had two adults, one son and one daughter, with whom he used to spend little time. Indeed, the owner established that the dog passed most of the day on the terrace, to prevent the animal's landlady from falling, since she suffered from motor problems. He used to go out twice a day with the owner and spend most of his time alone on the balcony, without any possibility to enter the house. The games usually played by the patient with the pet owner consisted of throwing a ball (predatory play, as chasing balls) and pull/spring (tug play) as well. The first symptoms arose around 1 year of age, when he started showing attitudes to chase moving cars. Therefore, the owner decided to contact one of the dog educators, whom to undertake a path with, although it was not finalized, because of no appreciable results for the owner. When the dog reached 2 years of age, the owner decided to repeat that experience, by employing a dog trainer with aversive methods. Such an educational program led the patient to live in a box inside the training center for 6 months, without seeing the owner during the first trimester, as requested by the trainer. The next 3 months, they scheduled to visit the dog once a week, with the aim to work with him, under the trainer's direction. The techniques used there mainly relied on the strangling of the collar and constraint, every time he made a mistake (when he pulled on a leash or hurled himself at cars). Upon going back home, the patient looked “quieter”, although he normally began to experience alterations in the sleep patterns and go around the table or chairs, especially in the presence of loud noises or barking. The owner did not report any other abnormal behavior, until the dog was retired for 2 weeks in the training center, where he had been previously. Unfortunately, on his way back the owner noticed a worsening of these symptoms. A few days later, he was taken to the groomer and, when he got back home, the owner reported episodes of TC behavior. At the same time, once he decided to adopt one more dog, namely, a 2-month-old female of medium-large breed, the health conditions of the patient did not ameliorate at all. Indeed, injuries became more evident since, although the dog was not allowed to see the puppy in the house, he started biting his tail and left thigh, so that contused wounds caused him to be taken and admitted to the veterinary emergency room (during the hospitalization, the patient was stabilized, carried in sedation, and treated for the surgical cleaning of wounds). During the behavioral examination, the patient showed a state of continuous agitation, hypersalivation, circling around the table, hyperventilation, hyper motricity, and panting.

**Table 1:** Correlation between the dog's age and situations during the dog's life.

**Age**	**Situation**
20 days	The dog was recovered in the countryside
20 days until 4 months	The dog was hosted by the volunteer
1 year	First aggressions against moving cars
1 year	First dog training with reward-based methods, for 3 months
2 years	Second dog training with aversive-based methods for 6 months
2 years and 6 months	Signs of anxiety in the dog
6 years	The dog is left 15 days in dog's kennel
6 years and 4 months	The dog has been in grooming
6 years and 5 months	The family adopted a new puppy; anxiety symptoms worsen
6 years and 6 months	The dog was admitted to the vet ER for tail injuries.

## Diagnostic Assessment

Compulsive disorder diagnosis requires to rule out all possible medical causes inciting such a behavior, including food intolerance, parasites, bone and joint problems, skin and neurological diseases (i.e., focal seizures), and other diagnoses considered (8). In the present work, CD diagnosis was based on the results of clinicopathologic testing and behavioral life history; physical, behavioral, and neurological evaluation; complete blood count and chemistry; abdominal ultrasound; and X-ray hip/tail morphology assessment (see Supplementary Materials). After the CD diagnosis was drawn, the veterinary behaviorist prescribed the therapeutic intervention, based on an interdisciplinary approach, including a pharmacological plus nutraceutical treatment, and behavioral rehabilitation, to be addressed in the patient. In particular, along with α-s1 casozepine (Zylkene^®^) 450 mg (2 tablets) BID given to him for 6 months, and fluoxetine 25 mg (2 tablets), SID for 6 months, the owner was given primary instructions to adequately manage the patient, relying on (a) the creation of a consistent and predictable environment and daily routine upon which the dog had control, in order to engage him in desirable activities (i.e., resting, perching, and playing with objects) and prevent undesirable behaviors; (b) consistent and predictable consequences that use rewards to encourage desirable behaviors rather than punishment, aiming at discouraging undesirable ones; (c) providing sufficient enrichment and outlets to meet the dogs, thus ensuring the owner's responses not to further reinforce or aggravate with anger, punishment, and agitation; (d) paying attention to the identification and removal (or reduction) of stressors that lead to the conflict, such as excessive manipulation and brush use; and (e) focusing on the reinforcement of desirable alternative behaviors to the compulsive attitude, including resting on a mat or chewing on an appropriate chew toy. According to pharmacological protocols, 15 days after fluoxetine and α-s1 casozepine treatment, the dog instructor started the rehabilitation therapy. In this respect, our goal was to increase the dog's wellbeing, by allowing him to implement collaborative, affiliative, social, exploratory, and scouting motivations, highly expressed in the subject. Firstly, a rehabilitation program was configured, by means of reward methods at the dog instructor center, which normally works in a systemic and relational approach, so that the dog and the owner could experiment with new activities in a setting, where the expression of problem behavior was not automatic. Notably, olfactory games were arranged as one of the main rehabilitation features. Accordingly, the dog was trained to perform an olfactory research, involving finding a treat in the dog's field, by following the verbal signal of “Search,” with the aim of lowering the state of emotional activation (arousal). In addition, dog socialization sessions were held with the newly adopted puppy, allowing them to spend some time at a dog field, or rather in nature. Furthermore, the training was accomplished at home, to increase the game attitude between dog and owner (see Supplementary Video), through homemade activities based on the dog's agility with reward, without competition or stress (i.e., passing under a bench, jumping over a small obstacle, following a slalom chair; see Supplementary Video). During the lessons, the owner was instructed to identify triggers, read facial and body language to be able to preempt the behavior, and direct him toward an acceptable one. The behavioral therapy session was weekly based for the first 3 months, becoming fortnightly during the last 3 months. Four follow-up sessions were carried out by the veterinary behaviorist (Table 2). In this respect, 15 days following fluoxetine and Zylkene^®^ administration, the first by-telephone medical examination reported, on the one hand, no gross fluoxetine-associated side effects in the dog, unless there was a slight inappetence in the first 5 days of the treatment, and on the other hand, an improvement in the duration and frequency of the TC symptom. Thus, from that time on, the rehabilitation program was allowed to start off. One month later, a second follow-up was performed during medical examination, and a significant improvement of the patient was found, who did not display any frustration and agitation symptoms at the time of the visit. Again, the owner reported a decreased reactivity when following barking of neighbors' dogs and the passage of cars as well. Three months after, during the third follow-up (by phone), the owner reported no new TC episodes, as the patient showed more cooperative and social skills—both with them and with other dogs. The final follow-up was performed at 6 months, based on the physical examination, where the veterinary behaviorist did not observe any skin lesions or tail chasing episodes, as reported by the owner. Interestingly, the owner was satisfied with the therapies carried out up to that moment and reported an easier dog management, even leading him to enter the house, especially in the very relaxing moment (evening) for the family.

**Table 2:** Timeline between the period of follow up and dog's behavior.

**Follow up by** **Vet behaviorist**	**Period**	**Way**	**Dog's behavior**
1°	15 days following pharmacological plus nutraceutical treatment	By phone	Slight inappetence, a reduction in symptom of tail chasing. *Star with behavioral rehabilitation therapy*
2°	After a month of starting the rehabilitation program	During medical examination	No symptoms of frustration and anxiety. Decreased reactivity of dog
3°	After 3 months starting the rehabilitation program	By phone	No more episodes of tail chasing. More collaborative and sociable dog
4°	After 6 months starting the rehabilitation program	During medical examination	No OCD symptoms. An easier dog management

## Discussion

TC is a classic OCD-like behavior experienced by dogs, generally occurs in bouts, and might include episodes in which animals stare at their tail quietly for a while, before resuming chasing. The existence of obsessive thoughts in animals is still a matter of debate, since obsessions by definition occur within the psyche and cannot be directly measured by an external observer. On the other hand, according to DSM-V criteria, compulsions correspond to repetitive behaviors that the individual feels driven to perform in response to an obsession, or according to rules that must be rigidly applied. Thus, unlike iterative mental acts, repetitive behaviors occurring in animals can be observed (9). Alongside a potential role of a breed-dependent genetic susceptibility upon CD onset and severity (5, 7, 10), more additional reasons for the occurrence of such a pathological behavior are thought to include nutritional status, conflict situations, attention-seeking attitude, and neurological and dermatological diseases (11). In the TC case here analyzed, we failed to find any clinical or neurological alterations, whereas other behavioral traits, associated with the medical history, including the anxiety state, conflict condition (due to the isolation in the terrace since puppyhood), past training with aversive stimuli, and departure from the family group, led us to draw the CD diagnosis (7). Recently, in the first large-scale quasi-experimental study of companion dog training (*n* = 92), de Castro et al. found that dogs trained with aversive stimuli displayed more stress-related behaviors, displayed higher cortisol levels, and were more “negative” in a cognitive bias task, when compared to the animals trained with either reward or mixed methods (12). These findings strongly suggest that using aversive stimuli during the training impairs the welfare of companion dogs, both inside and outside the educational context. In parallel, one of the studies aimed at assessing the link between training methods and dog–owner relationship showed that a secure attachment seems to be more effective in dogs trained with reward methods, as revealed by behaviors observed during the “Strange Situation Procedure” (13). Therefore, in the present study we documented a TC case in a 7-year-old intact male German Shepherd mixed-breed dog, who was managed by means of an interdisciplinary strategy, based on nutraceutical, pharmacological, and behavioral approaches. Interestingly, we observed (1) a significant TC reduction in the dog, in terms of frequency and duration of attacks; (2) an improvement in the ability of the patient to socialize with humans and other dogs, as well; (3) a robust reduction of anxiety-related symptoms occurring before starting the combined treatment; and (4) an overall welfare of the dog and, notably, an easier patient management by the owner.

### The Value of Socialization During Rehabilitation

Together with primary and juvenile phases, the socialization period (also known as sensitive period) in dogs is regarded as a key time window (3–14 weeks of age) to the development and maintenance of long-lasting human relationships, since they start learning how to interact with their mothers and littermates and cope with stressful events (14, 15). Accordingly, young puppies rear in isolated environments, frequently engage in “self-play” (i.e., TC), and are likely to display abnormal behaviors. Therefore, the sale of puppies, which either are weaned earlier or live alone in small, isolated places, may contribute to the development of canine TC and, in some cases, stereotypic behaviors (7, 16, 17). Based on this, we reasoned on the importance to point toward the increase of the socialization experiences in patients with family and other dogs living in the same context. In particular, the cohabiting dogs who interact at the field for the first time, and share olfactory games, try to hang around in an extra-urban environment, while take some rest in the house.

### The Importance of the “Unconventional” Integrated Therapy

Anxiety represents a further potential TC-related feature, since the tricyclic antidepressant, clomipramine, and fluoxetine, one of the selective serotonin reuptake inhibitors (SSRIs), are equally effective to treat anxiety and compulsive and aggressive behaviors in CD patients (18–20). Irimaiiri and coworkers reported that, despite equivocal results, fluoxetine administration at a therapeutic dose might be effective in treating 31 CD dogs, with few documented side effects, including lethargy and decreased appetite, which occurred in a small number of animals (21). Therefore, based on the previous findings showing that fluoxetine is able to restore thought and action, reduce the dog's impulsivity, and cause reflection before acting (22), we decided to use fluoxetine in our clinical case. For the same reason, we administered as add-on therapy α-s1 casozepine, a non-pharmaceutical compound that seems effective to counteract anxiety-related behaviors in dogs and other species (22, 23). The bioactive peptide α-s1 casozepine is the main cow's milk protein, characterized by the ability to bind to the GABA_A_ receptor (24), which is widely used as a pharmacological target for benzodiazepine-type compounds to treat epilepsy, insomnia, anxiety, and panic disorder (25). In this respect, several studies showed that α-casozepine has both anxiolytic and sleep-promoting properties in different species, including humans (26–29). Moreover, studies in rats showed that the anxiolytic effect of α-s1 casozepine is comparable to that of diazepam, except for the fact that it did not induce a disinhibition state, typically observed after benzodiazepine ingestion in humans (30).

### Why to Choose an Interdisciplinary Approach?

As reported by Overall and Dunham, the combination of behavioral modification and medication led to a significant decrease in intensity and frequency of CD in most animals (19). Moreover, this approach is often indicated as a choice to deal with behavioral problems and addresses the wellbeing of the pet, as previously described by Landsberg's studies (8). In addition, in a recent paper by Mills and colleagues, about the separation-dependent anxiety, the authors took advantage of a behavioral modification program combined with fluoxetine treatment, and found a more optimistic attitude in the patients, along with their general mood and behavioral improvement, when compared to the control group (31). Interestingly, our data are in line with those from Powell and coworkers, who showed that the success (or failure) of the behavioral rehabilitation for the dog is not only based on the physical and mental state of the animal but also on the role that the personality of the human companion normally plays during the rehabilitation program (32). The dog instructor and the veterinary behaviorist could provide a valid support to create a relaxed atmosphere more easily, by using a more specific and tailored strategy to better cope with and manage TC disorder. Creating the conditions so that the dog's owner can be positive and opens to the experience, listen to him, and accompany him, instead of judging and rebuking, represents a prerequisite for a tight connection between dog–owner and dog–behaviorist as well. Therefore, it is thought to be extremely important the specific formation of the instructors who collaborate with the veterinary, and that is why in Italy there is a difference between dog educator, dog trainer, dog instructor, and veterinary behaviorist. In this respect, according to the Italian legislation (UNI 11790:2020), a dog educator normally works with his own technical competence to guide the human–dog interaction, by implementing educational programs shedding light on a solid familiar relationship and social coexistence. On the other hand, the dog instructor (also known as the Italian acronym ESCAC) must be very good at analyzing and understanding even the pathological behavior in dogs and, if any, to adopt change programs, together with the veterinary behaviorist. A dog trainer develops specific skills and performances in the dog, through certain disciplines, like a coach. A veterinary expert in behavior with a degree in veterinary medicine has to follow both a theoretical and practical path of specialization, thanks to which he/she is able to diagnose behavioral pathologies and establish a prognosis, thus identifying objectives and useful times for rehabilitation therapy (33).

### The Importance of Cooperative and Olfactory Learning During Rehabilitation

As previously hypothesized, dog TC might arise, at least in part, from lack of activities, exercise, or stimulation (34), and increased arousal/frustration and boredom were well-described as one of main CD-triggering factors (7, 34, 35). To this end, cooperative activities between dog and owners are considered of great importance in the rehabilitation field (36). Thus, domestic dogs, like children, seem more prepared to use human gestures when they are given cooperatively, thus ending up reducing stress. Notably, our therapeutical approach here described is in line with what suggested by Horowitz and colleagues, about the importance of employing more sessions based on olfactory learning in the rehabilitation program, considering the excellent sense of smell in dogs (37). In our opinion, it could be possible that these great olfactory qualities are satisfied during these sessions. Of note, one of the main neurotransmitters associated with the functional sense of the smell is dopamine, which is able to modulate the olfactory tubercle activity in the olfactory cortex (where it plays a role in the overall “reward” system of the brain) and eventually the periglomerular cells in the olfactory bulb (38). Therefore, olfactory research increases concentration and leads the dog to an intermediate level of arousal, which is the most appropriate parameter for learning and integrating the subject in the social context (22).

### Limitations of the Study

The present case study represents a proof of concept about the positive impact of employing an integrated therapeutic strategy, made up of conventional treatment (fluoxetine), associated with both α-casozepine administration and a thoughtful behavioral program, performed by a veterinary behaviorist together with a dog instructor. However, we followed up on the patient until 6 months since we started the therapy; thereafter, we have not heard from the owner about the condition of his dog. This represents an important issue, as well as the advanced age of the patient, since we cannot rule out a putative biased approach in the way we managed him during the onset and severity of the pathology. Therefore, finding a shorter and better recovery time in the German Shepherd mixed-breed dog might pave the way for further studies employing different cohorts of animals of different breeds, age, and sex, to enlarge and, maybe, make more reliable this “unconventional” approach in the treatment of OCD-like disorders.

## Conclusions

Compulsive behaviors in dogs share clinical similarities with human OCD, including repetitive nature, early onset, and the ability to respond to medications, based on SSRI prescriptions. Consequently, canine compulsive behaviors have been suggested as a promising model for human OCD, with a good face and predictive validity (39). The interdisciplinary approach addressed in the present study could be helpful in the treatment and management of such a disorder for different reasons, as previously shown (40). First, it aids the therapeutic alliance with the family group, since it allows the clinician to show scientific rigor and confidence in the prediction of action (hence the importance of very precise knowledge of the drug that you want to use). Second, it immediately improves the emotional state of the patient, thus reducing the suffering state related to anxiety, fear, depression, and so on. Third, this strategy enhances the relationship with the family group because, considering the effect of emotional osmosis, emotions prevailing in the group will be more positive, once the pathological state of the patient has been reduced. Finally, it facilitates behavioral therapy, by allowing the reappearance of some plasticity attitudes, usually impaired in patients with behavioral diseases.

## Data Availability Statement

The raw data supporting the conclusions of this article will be made available by the authors, without undue reservation.

## Ethics Statement

Ethical review and approval were not required for the animal study because this was a retrospective case report. Written informed consent was obtained from the owner for the publication. Written informed consent was obtained from the owners for the participation of their animals in this study.

## Author Contributions

Dd'A, LS, FN, and CG contributed to the conception and design of the study and wrote sections. Dd'A, LS, FN, LA, and RC wrote the first draft of the manuscript. All authors reviewed and approved the final submitted manuscript.

## Conflict of Interest

The authors declare that the research was conducted in the absence of any commercial or financial relationships that could be construed as a potential conflict ofinterest.

## Publisher's Note

All claims expressed in this article are solely those of the authors and do not necessarily represent those of their affiliated organizations, or those of the publisher, the editors and the reviewers. Any product that may be evaluated in this article, or claim that may be made by its manufacturer, is not guaranteed or endorsed by the publisher.
