# Supplementary material for: An Interdisciplinary Approach for Compulsive Behavior in Dogs: A Case Report
Source: Front Vet Sci. 2022 Mar 24;9:801636. doi: 10.3389/fvets.2022.801636 (PMC8988433; doi:10.3389/fvets.2022.801636)

**ECC report**


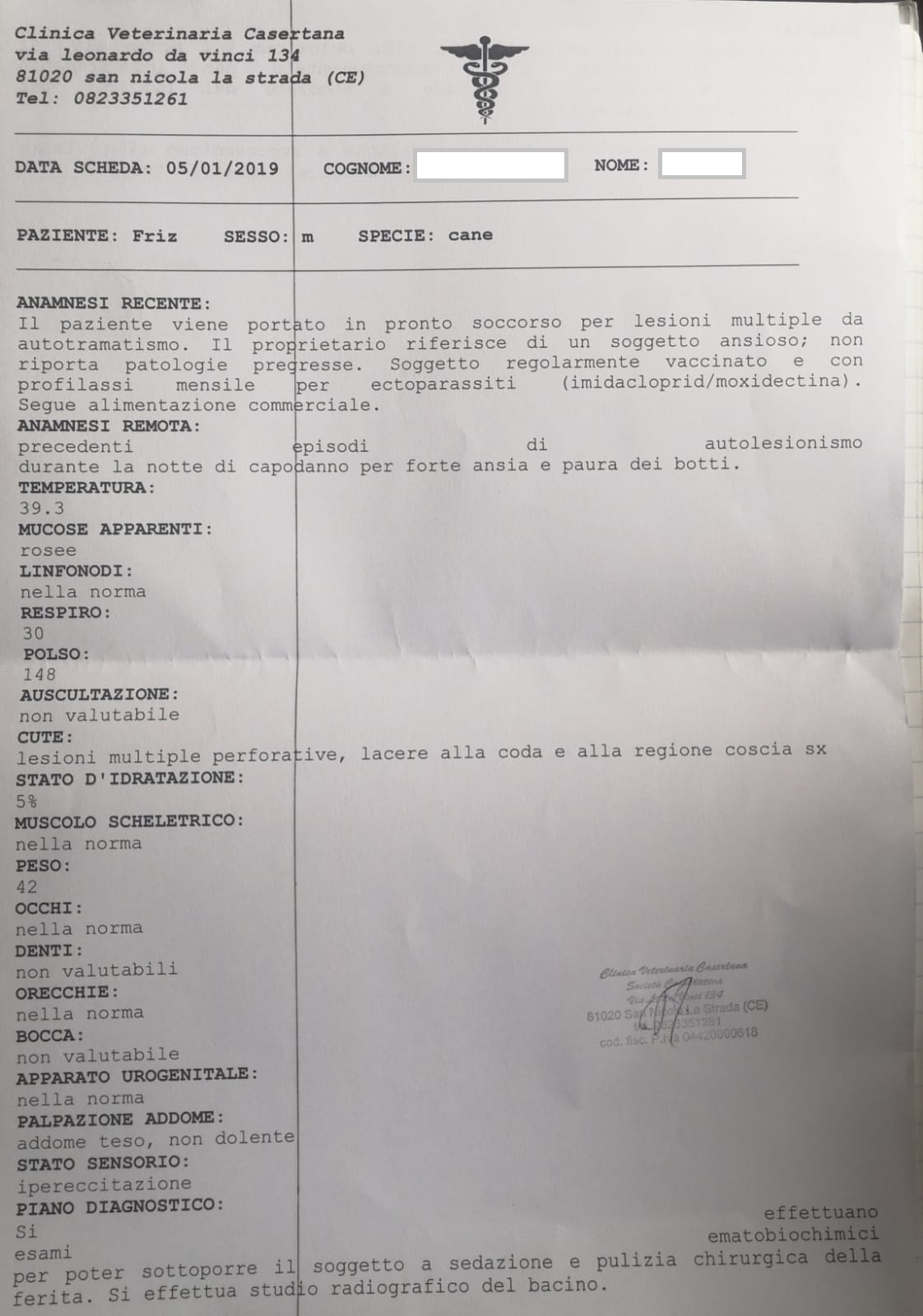


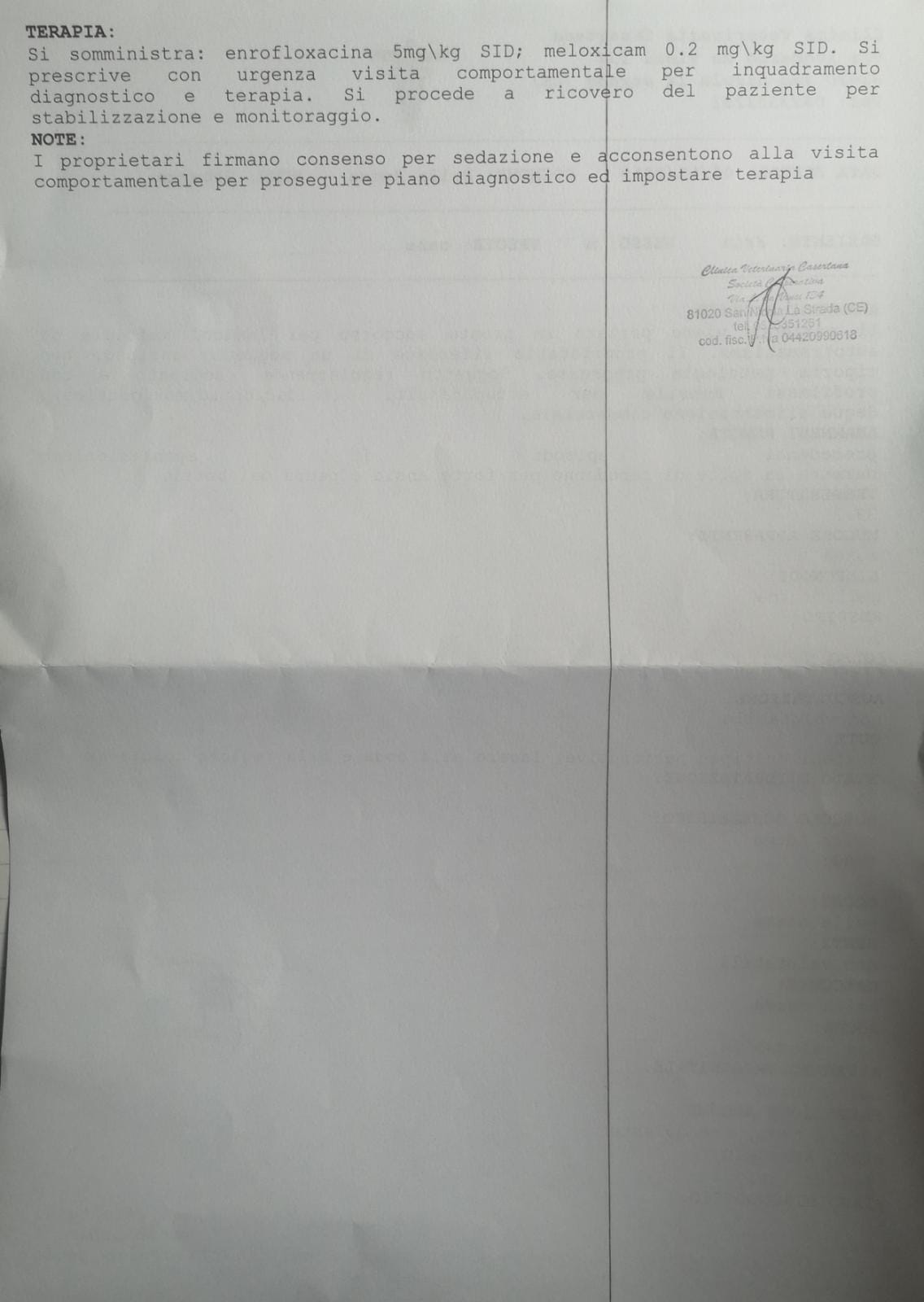


**Biochemistry test in ECC**


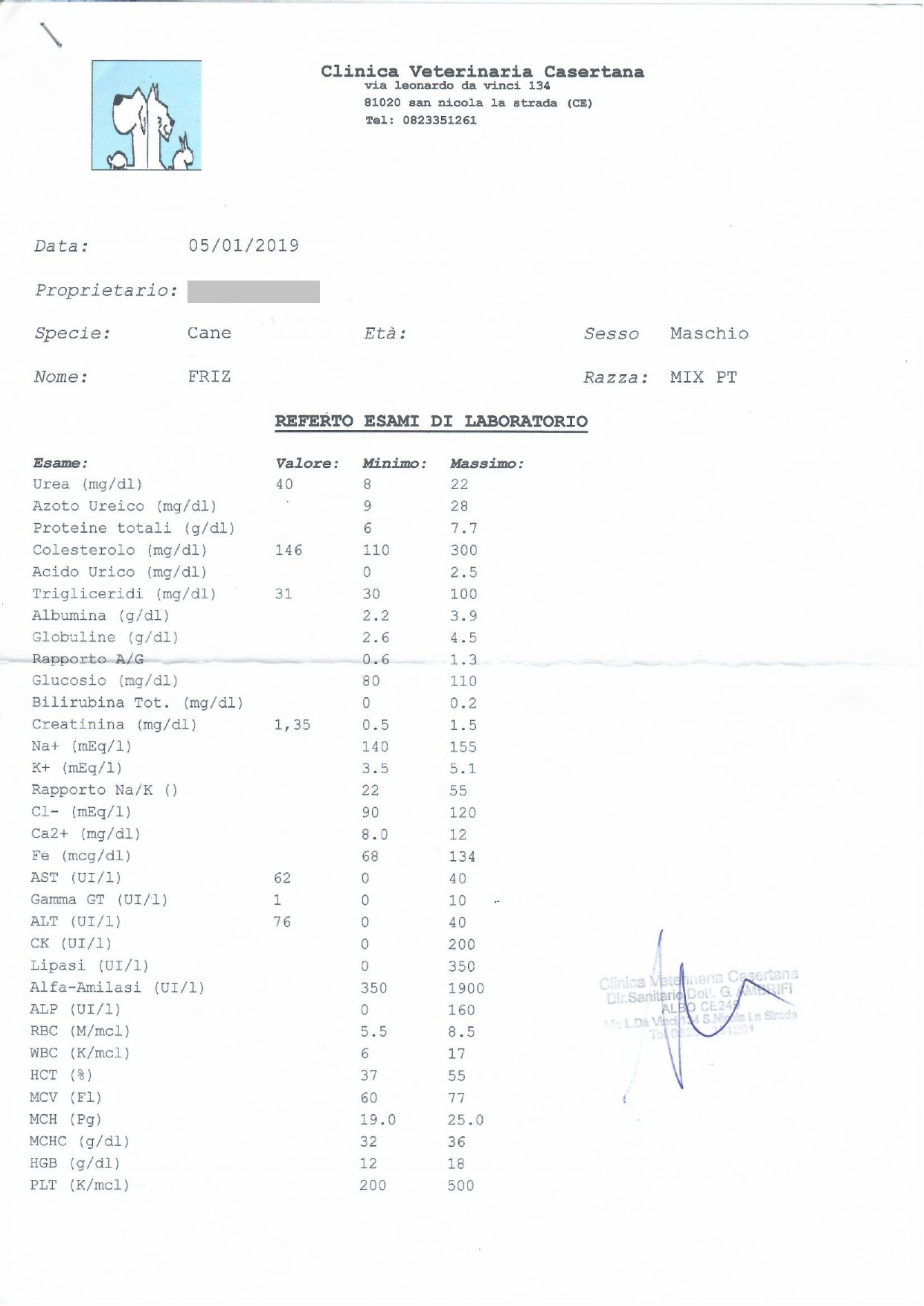


**CBC test in ECC**


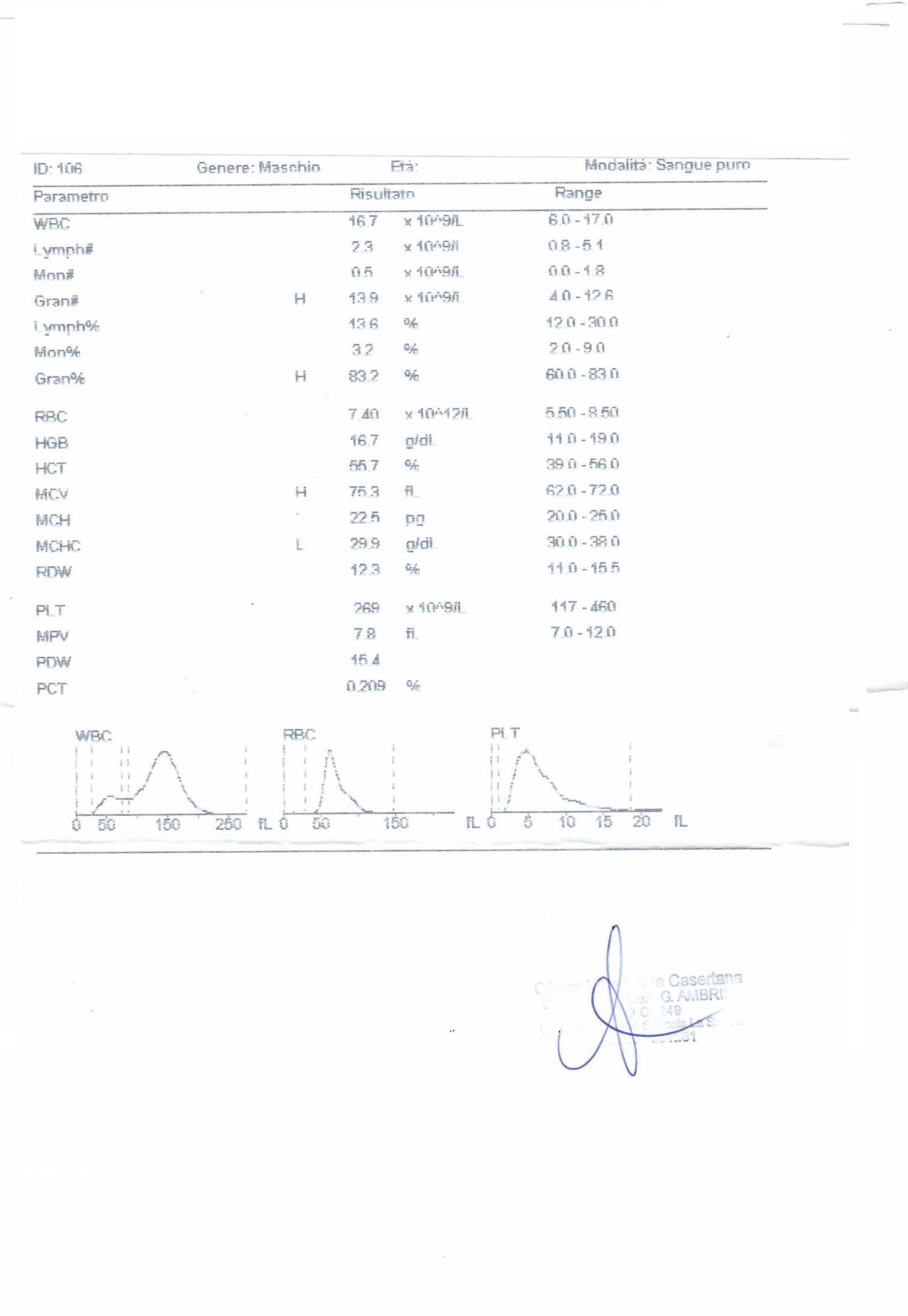


**The results of the ultrasound abdomen, required by the behavioral veterinarian, for diagnostic assessment of CD. Ultrasound** **carried out few days after taking charge of the patient by the behavioral vet.**


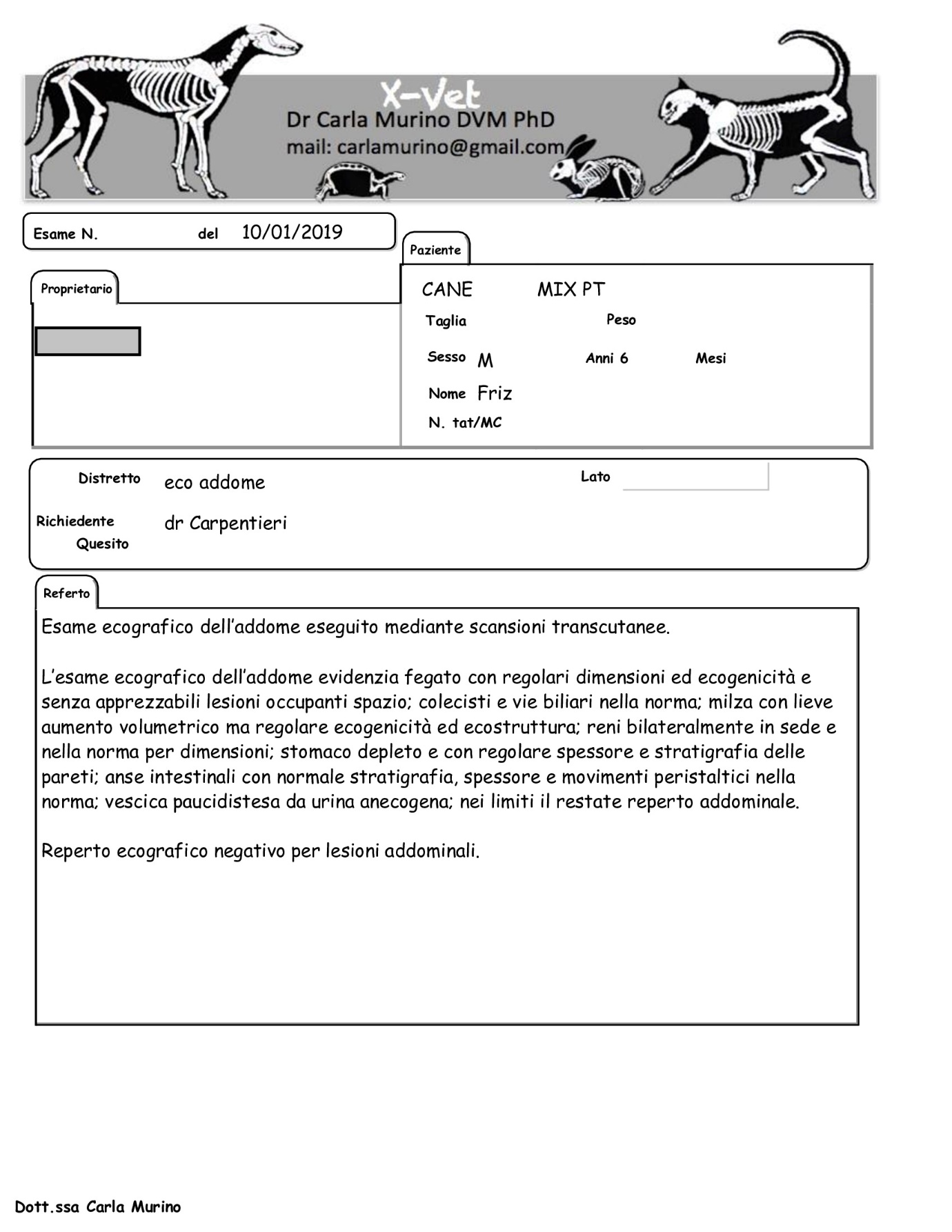


**The results of the X-ray of tail and hip, when dog was assisted in ECC .**


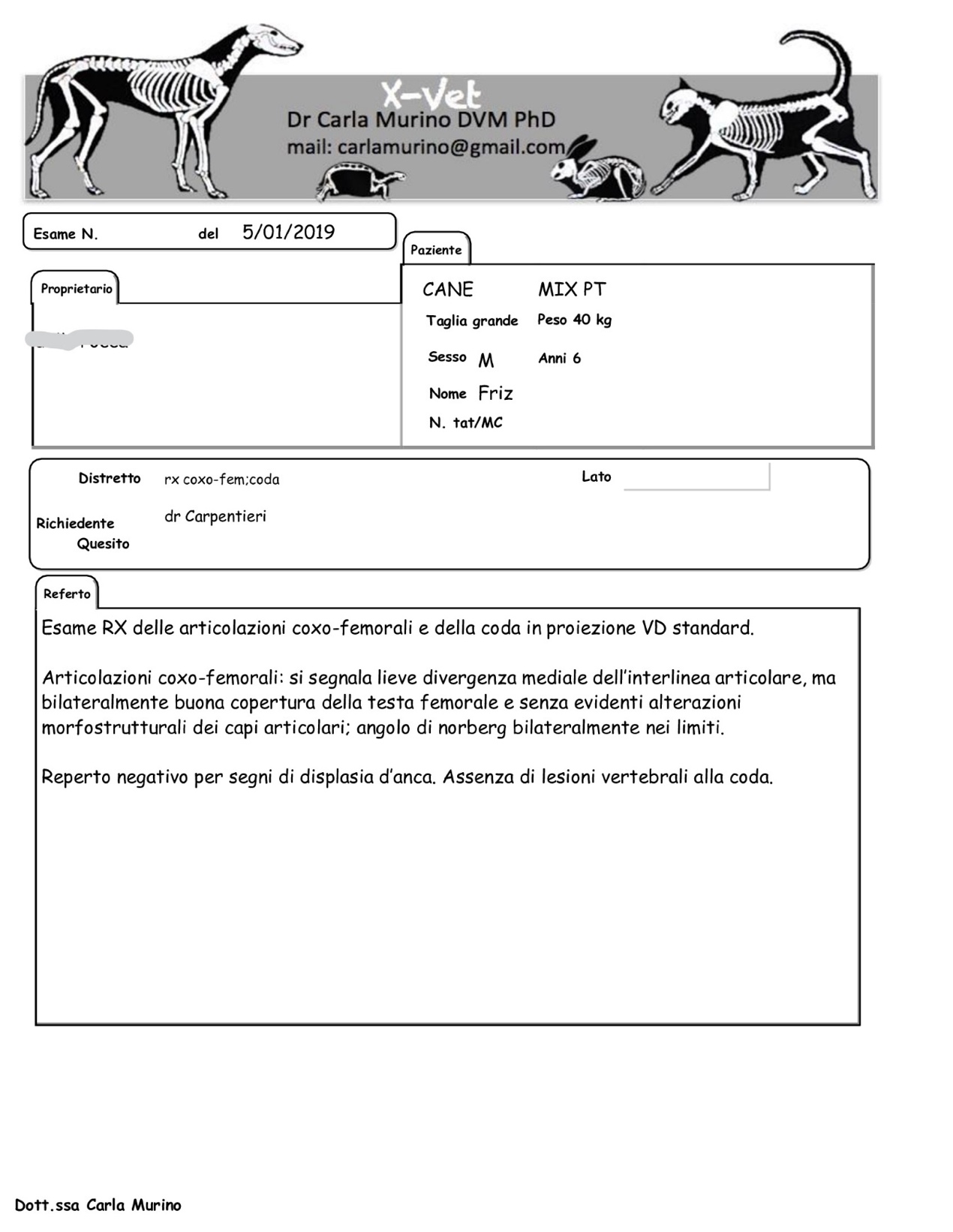

Supplement: Supplementary file 1 [file Data_Sheet_1.ZIP › d'Angelo et al 2022-Supplementary Materials/d'Angelo et al 2021-Supplementary materials.docx]
